# Combining the KRAS^G12C^ inhibitor adagrasib with anti-PD-1 immunotherapy improves overall survival and prevents recurrence in preclinical models of brain metastasis

**DOI:** 10.1093/noajnl/vdag107

**Published:** 2026-06-02

**Authors:** Consuelo Torrini, Naema Nayyar, Gregory R Wojtkiewicz, Anita Giobbie-Hurder, Magali A de Sauvage, Christian Migliarese, Elizabeth J Summers, Nazanin Ijad, Erika Yamazawa, Britney S Zhang, Emily M Sullivan, Varun Sasisekharan, Leland G Richardson, Braxton M Marion, Peter Olson, Hiroaki Wakimoto, Priscilla K Brastianos

**Affiliations:** Krantz Center for Cancer Research, Massachusetts General Hospital, Boston, Massachusetts, USA; Krantz Center for Cancer Research, Massachusetts General Hospital, Boston, Massachusetts, USA; Krantz Center for Cancer Research, Massachusetts General Hospital, Boston, Massachusetts, USA; Krantz Center for Cancer Research, Massachusetts General Hospital, Boston, Massachusetts, USA; Krantz Center for Cancer Research, Massachusetts General Hospital, Boston, Massachusetts, USA; Department of Neurosurgery, Massachusetts General Hospital, Boston, Massachusetts, USA; Krantz Center for Cancer Research, Massachusetts General Hospital, Boston, Massachusetts, USA; Krantz Center for Cancer Research, Massachusetts General Hospital, Boston, Massachusetts, USA; Krantz Center for Cancer Research, Massachusetts General Hospital, Boston, Massachusetts, USA; Department of Neurosurgery, Massachusetts General Hospital, Boston, Massachusetts, USA; Krantz Center for Cancer Research, Massachusetts General Hospital, Boston, Massachusetts, USA; Mirati Therapeutics, Inc., A Bristol Myers Squibb Company, San Diego, California, USA; Department of Neurosurgery, Massachusetts General Hospital, Boston, Massachusetts, USA; Krantz Center for Cancer Research, Massachusetts General Hospital, Boston, Massachusetts, USA; Divisions of Hematology/Oncology and Neuro-Oncology, Massachusetts General Hospital, Harvard Medical School, Boston, Massachusetts, USA

**Keywords:** anti-PD-1, brain metastases, immunotherapy, KRAS inhibitor, targeted therapy

## Abstract

**Background:**

Despite advances in treatment, brain metastasis (BM) management remains a significant challenge. Adagrasib is a brain-penetrant KRAS^G12C^ inhibitor active in patients with BM. KRAS mutations are linked with immune escape and may contribute to the limited clinical benefit from single-agent immune checkpoint inhibitors (ICI) targeting PD-1/PD-L1 in BM. Although adagrasib sensitizes extracranial tumors to ICI, its intracranial benefit combined with immunotherapy remains unknown. Here, we evaluate adagrasib with ICI in mouse models that mimic the BM immune microenvironment.

**Methods:**

We tested the in vitro efficacy of adagrasib on two Kras^G12C^-mutant murine cancer cells: colorectal CT26^G12C^ and lung cancer KPAR^G12C^. Murine BM models resembling the immunologic characteristics of BM were established by subcutaneous and intracranial injection of these cells. Animals were treated with adagrasib combined with anti-PD-1 and monitored for intracranial tumor growth and survival. Disease-free mice after 11-13 weeks were rechallenged with a higher tumor cell dose to assess tumor-specific memory.

**Results:**

Three-week adagrasib monotherapy and combination therapy with ICI demonstrated benefit in colorectal and lung cancer BM models. Adagrasib alone and in combination demonstrated similarly potent anti-tumor effects against extracranial tumors. While monotherapies reduced intracranial tumor growth, adagrasib with ICI showed the most favorable outcome. Although both adagrasib monotherapy and combination therapy extended survival, long-term intracranial disease control after rechallenge was the greatest with combination therapy.

**Conclusions:**

Adagrasib with ICI improved long-term survival and blocked CNS progression in dual extra- and intracranial BM models.  These findings support investigation of adagrasib with ICI in patients with KRASG12C-mutant BM.

Key Points

- KRAS-targeted therapy combined with anti-PD-1 immunotherapy was tested in dual extracranial and intracranial cancer models.
- Anti-PD-1 immunotherapy extends the efficacy of KRAS-targeted therapy in preclinical models of intracranial metastatic lung and colorectal cancer.

Importance of the Study

Our data demonstrate that combining adagrasib with anti–PD-1 immunotherapy is an effective therapeutic strategy for controlling tumor growth and preventing BM recurrences in two preclinical models. Our findings support ongoing clinical investigation of ICI in combination with KRAS G12C targeted therapy for patients with BM with KRASG12C mutation.

Brain metastases (BM) affect approximately 200,000 patients in the US every year,^1^ and the numbers are rising with improvements in systemic cancer treatment. Despite therapeutic progress, BM are associated with significant morbidity and mortality, often causing debilitating symptoms such as seizures, cognitive decline, and severe headaches. In addition, current treatment options are limited, and many patients are excluded from clinical trials due to poor treatment tolerability and limited life expectancy, highlighting the urgent need for more effective and better-tolerated therapies in this setting.

Kirsten rat sarcoma virus oncogene homolog (KRAS) is one of the most frequently mutated genes in cancer,^2^ with alterations found in 86% of pancreatic adenocarcinoma, 41% of colorectal cancer, and 35% of lung adenocarcinoma patients.^3^ In addition, approximately 40% of lung cancer patients harboring KRAS mutations develop BM,^4^^,^^5^ underscoring the relevance of this genetic mutation in CNS (central nervous system) tumor progression. The predominant mutated site is the glycine in position 12, with frequent amino acid substitutions to cystine (G12C) or to aspartate (G12D).^6^

Recently, several compounds targeting the hyperactivated status of this protein have been developed, such as sotorasib^7^ and adagrasib,^8^ which block the activity of mutant KRAS^G12C^. Importantly, preclinical studies demonstrated the efficacy of single agent adagrasib in colorectal cancer as well as in non-small cell lung cancer (NSCLC).^5^ Adagrasib can cross the blood brain barrier, which makes this agent of particular interest for clinical translation in patients with intracranial progression of KRAS^G12C^ mutant cancers. The encouraging preclinical results led to the development of clinical trials evaluating the efficacy of adagrasib in patients with metastatic colorectal cancer and non-small lung cancer (KRYSTAL-1 phase II trial, NCT03785249). The efficacy of adagrasib led to accelerated FDA approval in 2022.

Mutated KRAS in tumor cells influences the tumor microenvironment (TME), inducing expression and release of chemokines and cytokines that promote tumor immune evasion.^9^ As shown in previous studies,^10^^,^^11^ KRAS mutant cells have a high expression of IL-8 and CXCL3, via suppression of IRF2, as well as CXCL5, which are all CXCR2 ligands, and can suppress anti-tumor immunity in preclinical models of colorectal,^11^ pancreatic,^10^ and lung cancers.^12^ Because KRAS mutation events promote tumor immune evasion with high levels of PD-L1,^13^ targeting IRF2 upregulation or CXCR2 suppression can effectively overcome anti-PD1 resistance in KRAS-mutant tumors.^11^

In addition, KRAS mutation induces the recruitment of myeloid cells, via chemokine secretion, such as CCL2, and promotes the proliferation and expansion of immunosuppressive myeloid cells in in vivo models of colorectal cancer.^11^ Moreover, KRAS mutant tumor cells express high levels of CD47+, an inhibitory signal preventing tumor cells from being detected and phagocytized by anti-tumor macrophages.^11^

On the other hand, higher  T cell infiltration is also associated with KRAS expression, underscoring the importance of CD8+ T cells in the context of KRAS mutation and supporting the combination of immune checkpoint inhibitors (ICI) and KRAS inhibitors in pancreatic cancer.^10^ Similar conclusions are also supported by preclinical research in lung cancer, in which inhibition of KRAS was associated with increased levels of cytotoxic  T cell activation and antigen-presenting cells (APCs).^14^ In this context, KRAS-targeted therapy conferred an increased sensitivity to ICI in extracranial mouse models of lung^5^^,^^14^ and colorectal cancer.^11^ Importantly, BM exhibit significantly more tumor-infiltrating lymphocytes (TILs) than normal brain tissue and primary CNS tumors,^15-17^ supporting the investigation of ICIs in combination with KRAS-targeted therapy in BM. However, limited preclinical BM models hinder in vivo testing of these treatments. Given the encouraging extracranial results and our immunotypic mouse models that recapitulate BM immunology, in this study, we investigated the efficacy of a combination strategy of adagrasib and an anti-PD-1 therapy in preclinical models of BM, and showed for the first time its long-term intracranial benefit in preventing tumor recurrence.

## Materials and Methods

Cell Culture: CT26^KRASG12C^ cells were gifted by Mirati Inc. and KPAR^KRASG12C^ were purchased from Cancer  Tools.^18^ Cells were tagged with firefly-luciferase-mCherry (FmC) using lentiviral transduction to generate CT26^KRASG12C^-FmC and KPAR^KRASG12C^-FmC. Cells were maintained in culture as described in the Supplementary Material.

Dose-response assay: CT26 and KPAR cell lines were cultured in 96-well plate and tested with different concentrations of adagrasib to identify its IC50. After 72 h incubation with the compound, cell viability was assessed as luminescence using the CellTiterGlo kit.

Animal Studies: All animal studies were approved by the IACUC at Mass General Brigham (MGB) and conducted on 6-8-week-old female C57BL/6 and BALB/c mice from Charles River Laboratories. Mice were housed at the MGB Center for Comparative Medicine animal facility in a 12-h light-dark cycle with unlimited access to food and water.

Immunotypic Models of BM: We evaluated anti-tumor immune responses using previously described immunotypic models of BM, where mice were implanted with dual subcutaneous and intracranial tumors.^19^^,^^20^ CT26^KRASG12C^ and KPAR^KRASG12C^ cell suspensions were injected subcutaneously (100,000 and 200,000 cells, respectively) three days before the intracranial tumor injections (50,000 CT26 cells; 100,000 KPAR cells). Cell doses were experimentally determined for each cell line, thus overall survival depends on the intracranial tumor growth. For tumor rechallenge, 200,000 and 400,000 cells of CT26 and KPAR, respectively, were injected in the left brain hemisphere, contralateral to the first tumor implantation. Intracranial tumor growth was monitored using bioluminescence imaging (BLI) at the MGB Center for System Biology Imaging Core as previously described.^21^

Drug Administration: Animals received 100 mg/kg adagrasib or vehicle orally twice a day (BID) for three weeks starting from 5 days after intracranial tumor injection. Anti-PD-1 or isotype control IgG was administered at 10 mg/kg intraperitoneally every 3 days.

Gene Expression: KPAR and CT26 cells were plated in 6-well plates and treated with adagrasib at 500 nM and 1 µM concentrations or DMSO for 24 h. RNA purified from the cells was used to evaluate gene expression via RT-qPCR using the TaqMan probes described in the Supplementary Material.

T cell isolation and downstream analysis: T cells were obtained and activated from C57BL/6 or BALB/c mouse spleens, adapting the published STAR Protocol.^21^ Spleens were mechanically dissociated and filtered to obtain single cells. Red blood cells were removed using the Gibco ACK Lysis buffer. The remaining cells were counted and plated in pre-coated wells with anti-CD3e and CD28 antibodies for T cell activation. After 24 h, T cells were expanded by adding IL-2 and then passaged every 2 days till day 7, when they were used in co-culture with CT26 or KPAR luciferase-tagged tumor cells at different dilutions (1:1 or 1:100) with adagrasib (500 nM), anti-PD-1 (10 µg/mL), or their combination. Tumor cell viability was assessed by the One-Step Luc Assay kit.

### Statistical Analysis

Statistical analyses used for each experiment are described in the figure legend. Generally, tumor growth experiments were analyzed using linear mixed models with log_10_-transformed tumor volume as the outcome.  The models included fixed effects for time, treatment group, and their interaction. Survival experiments were analyzed using Kaplan-Meier method. Multiple pairwise comparisons were adjusted using  Tukey-Kramer or Benjamini-Hochberg methods. SAS 9.4 and GraphPad Prism v.10.6.1 were used for statistical analyses.

There are no deposited data associated with this manuscript.

## Results

### Efficacy of the KRAS^G12C^ Inhibitor Adagrasib, in Combination With Anti-PD-1 Immunotherapy in an Immunotypic Mouse Model of KRAS^G12C^ Colorectal Cancer BM

**Figure 1.**
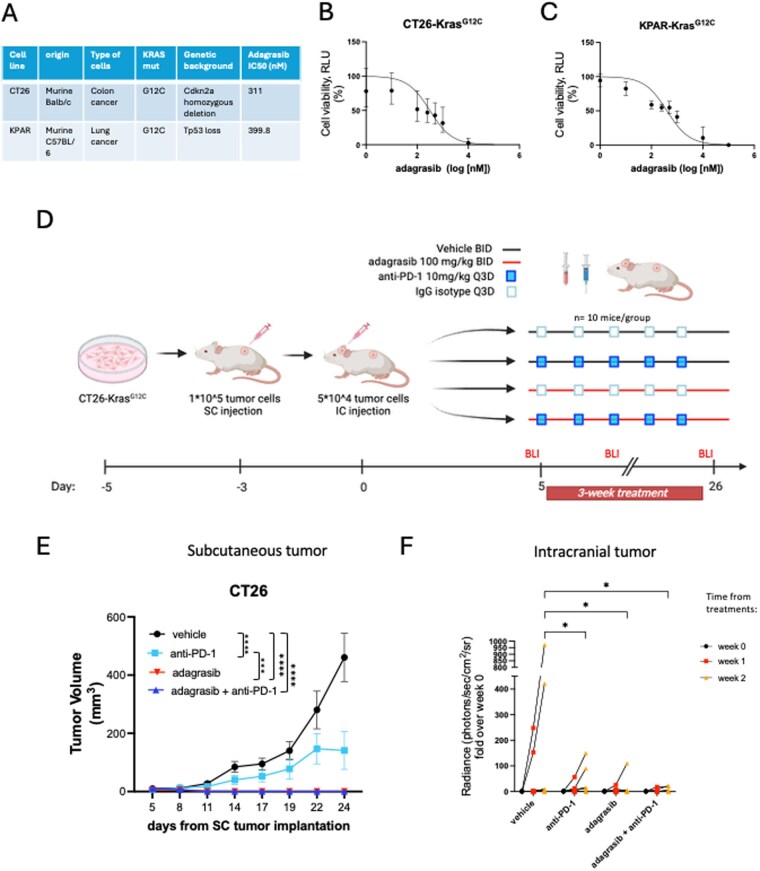
Adagarsib efficacy in vitro and in an in vivo model of colorectal cancer BM. (A) The table describes genetic background, origin, and IC50 of the tested cell lines. (B and C) Dose-response assay for the KRASi, adagrasib. CT26 colorectal cancer and KPAR lung cancer cells were tested to identify IC50 measuring cell viability. (D) Schematic of an immunotypical mouse model of colorectal BM. Created in BioRender. Torrini, C. (2026) https://BioRender.com/zfpsget. CT26 cells were implanted both subcutaneously and after 3 days intracranially. Shown is the 3-week adagrasib treatment schedule in combination with ICI. (E) Subcutaneous tumor volume measurements are shown over time. Statistical analysis was performed at the last timepoint and detailed in the table. Two-way ANOVA, Turkey’s multiple comparison test (***= *P* < .0002, **** *P* < .0001, *n* = 10 per group). (F) Intracranial tumor volume was monitored at 3 different time points, pre-, week 1 and week 2 of treatment, using BLI. Measurements are expressed in radiance. Statistical analysis was performed at the 2-weeks timepoint. Two-way ANOVA, Turkey’s multiple comparison test was used for statistical analysis (*= *P* = .05, *n *= 10 per group).

To evaluate KRAS^G12C^ targeted therapy in combination with anti-PD-1 immunotherapy, we identified murine cell lines that harbor the KRAS^G12C^ mutation, including the colorectal cancer cells, CT26-^KrasG12C^ and the lung cancer cells, KPAR^KrasG12C^ (Figure 1A). We first tested their sensitivity to the selective KRAS^G12C^ inhibitor, adagrasib, *in vitro* using CellTiterGlo assay. The dose-response assay demonstrated that both cell lines have a similar sensitivity to this compound, with an IC_50_ of 311 nM in CT26 cells and 399.8 nM in KPAR cells, respectively (Figure 1A-C).

We then evaluated whether adagrasib, at two different concentrations (500 nM and 1 µM), altered gene expression in a set of immune-related genes in CT26 and KPAR cell lines. We confirmed downregulation of KRAS-dependent MAPK targets following adagrasib treatment and found upregulation of MHC class I genes as well as cytokines, including Cxcl10, in both models (Supplementary Figure S1A and B). We detected increased transcript levels of Cd274 encoding PD-L1 and receptor tyrosine kinase Axl only in KPAR cells, suggesting that adagrasib treatment may promote a more suppressive tumor immune microenvironment in the KPAR model (Supplementary Figure S1B).

Next, we assessed the efficacy of adagrasib in combination with anti-PD-1 therapy in syngeneic mouse models that mimic BM immunology (immunotypic models) using BALB/c or C57BL/6 mice. We generated BM models that involve subcutaneous (SC) and intracranial (IC) tumor implantation to recapitulate the immunologic features in BM.^19^^,^^20^ First, we established a CT26 dual subcutaneous intracranial model of BM, injecting CT26-Kras^G12C^ cells SC, followed by IC implantation of CT26-Kras^G12C^-FmC tagged cells three days later. All treatments were initiated 5 days after intracranial tumor injection (Figure 1D). Adagrasib was administered twice a day (BID) via oral gavage at 100 mg/kg for three weeks, and anti-PD-1 antibody was injected intraperitoneally every three days (Q3D) at 10 mg/kg for five doses total. We monitored mice for body weight, which remained constant during the entire treatment period, suggesting good tolerability of the treatments (Supplementary Figure S2A). In addition, subcutaneous and intracranial tumor growth were assessed, quantifying subcutaneous tumor volume and intracranial bioluminescence (BLI), respectively (Figure 1E and F and Supplementary Figure S2B). Although mice receiving anti-PD-1 presented with significantly smaller SC tumor volume compared to those receiving vehicle (control group), tumor growth was completely abrogated in mice treated with adagrasib alone or in combination with anti-PD-1 (Figure 1E and Supplementary Figure S2B-E). Bioluminescence imaging showed similar anti-tumor effects intracranially, with a trend for the greatest benefit with the combination treatment (Figure 1F and Supplementary Figure S2F).

We performed an additional BLI follow-up at 3 weeks post-treatment (Supplementary Figure S2G). Seven out of 10 animals were intracranial tumor-free in the adagrasib-only and the combination groups, as compared with 5 out of 10 and 1 out of 10 animals in the anti-PD-1 group and vehicle group, respectively.

### Treatment Responders Rejected Intracranial Rechallenge With CT26 Tumor Cells

**Figure 2.**
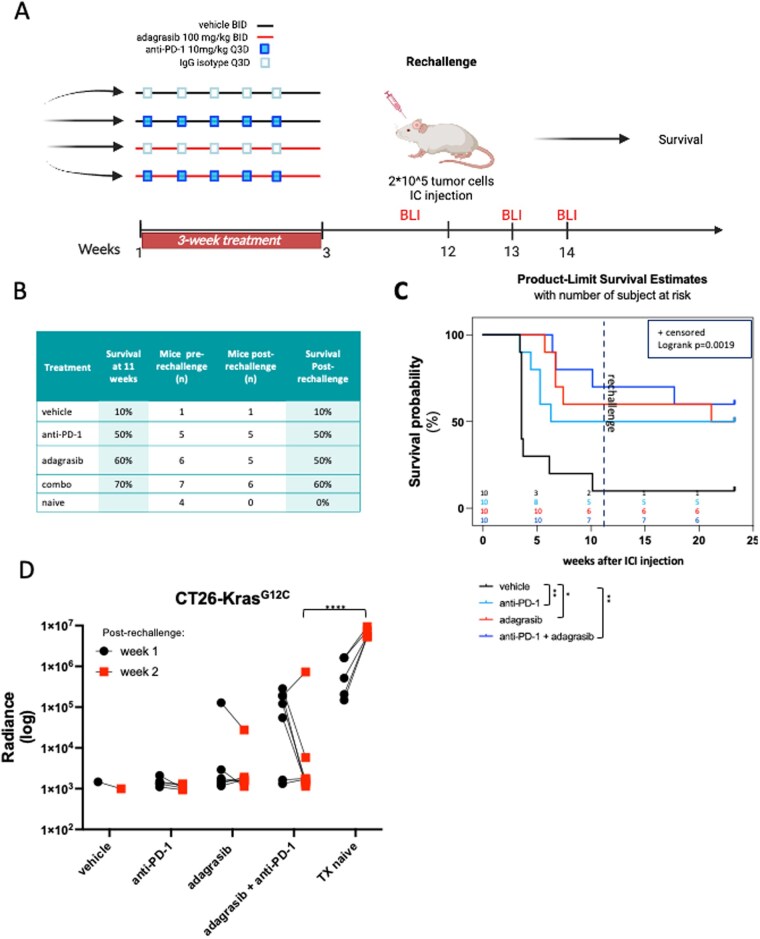
In vivo CT26 tumor rechallenge. (A) Schematic of in vivo CT26 tumor rechallenge. Created in BioRender. Torrini, C. (2026) https://BioRender.com/o598tcz. Survived animals from each group were rechallenged with a 5 times higher concentration of tumor cells and monitored for overall survival and tumor growth using BLI. (B) Table shows the census of surviving animals at 13 weeks (pre-rechallenge), mice enrolled in the rechallenge experiment, as well as the survival post-rechallenge. (C) Animal overall survival was evaluated at 22 weeks after IC injection using log-rank test (*P* = .0019). Pairwise comparisons were used with the Benjamini-Hochberg adjustment (** *P* < .01, *P = .05) (*n* = 10). (D) Intracranial tumor volume was monitored at two time points post-rechallenge using BLI. Statistical analysis assessing the significance of tumor volume at week 2 compared to that observed one week after rechallenge for each group (Two-way ANOVA analysis using Šídák’s multiple comparisons test, ****P < .0001).

Because of the observed CNS tumor regression, we continued long-term animal follow-up (Figure 2A). We assessed the long-term efficacy of the treatments with overall survival (Figure 2B and C) and intracranial tumor growth (Supplementary Figure S3A, week 0) at the 9-week timepoint after treatment discontinuation. We observed that, in the vehicle group, only 10% of the animals were still alive, whereas in the anti-PD-1 group, 50% survived; in the adagrasib and combination groups, 60% and 70% of animals survived, respectively (Figure 2B and C).

To determine whether treatments induced anti-tumor immune memory in long-term responders, we rechallenged the tumor-cleared mice, injecting the same tumor cells in the opposite brain hemisphere at a cell number four times higher than the original implantation (Figure 2A). A group of 4 mice that had never been injected with tumor cells (tumor naïve) served as a control for tumor growth. We monitored intracranial tumor growth using BLI (Figure 2D and Supplementary Figure S3A). As expected, after 2 weeks from the rechallenge, all the tumor naïve mice died (Supplementary Figure S3B), due to rapid tumor growth (Supplementary Figure S3A). In contrast, most of the rechallenged mice that rejected the original implantation remained alive with stable tumor regression during the 12-week follow-up period after the rechallenge (Figure 2B and C). More specifically, in both anti-PD-1 and adagrasib monotherapy groups, 50% of the animals survived, in the combination therapy group, 60% were still alive, and in the control group, only 10% were alive.

Together, these data suggest that adagrasib alone or in combination with immunotherapy can provide a statistically significant long-term benefit in overall survival and prevent long-term intracranial recurrences in BM derived from CT26 colorectal cancer cells.

### Adagrasib Showed Intracranial Benefit in Combination With Anti-PD-1 Therapy in an ICI-Resistant KRAS^G12C^ Lung Cancer BM Model

**Figure 3.**
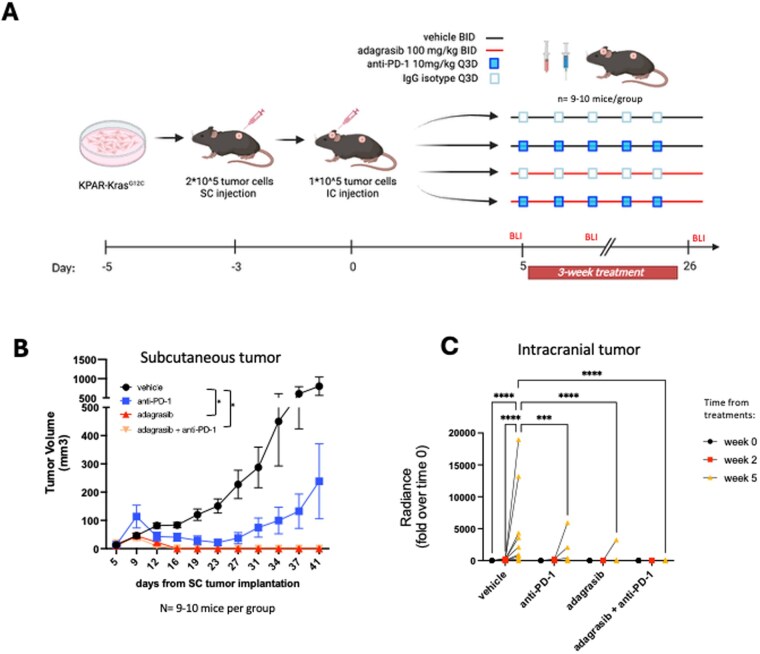
Lung cancer model of BM. (A) Schematic of an immunotypical mouse model of lung cancer BM. Created in BioRender. Torrini, C. (2026) https://BioRender.com/ggywwm5. KPAR tumor cells were implanted both subcutaneously and intracranially after 3 days. Shown is the 3-week adagrasib treatment schedule in combination with ICI. (B) Subcutaneous tumor volume measurements are shown over time. Statistical analysis was performed at the last timepoint. Two-way ANOVA, Turkey’s multiple comparison test was used for statistical analysis (*n* = 9/10 animals per group). (C) Intracranial tumor volume was monitored at 3 different time points, pre-treatment, and during treatment (week 2 and week 5), using BLI. Statistical analysis was performed using a Two-way ANOVA, Turkey’s multiple comparison test at 5 weeks: ****P < .0001, *P* < .001. *n* = 9-10 per group.

Because CT26 cells are known to be relatively immunogenic, we tested adagrasib in combination with anti-PD-1 therapy in a model established with a KRAS^G12C^-mutant cancer cell line, KPAR, which was reported to be ICI-resistant.^14^^,^^18^ We injected KPAR-^KrasG12C^ cells in C57BL/6 mice to generate an immunotypic BM model and evaluated the same therapies as described above (Figure 3). Here, we used the same drug administration protocol and readouts adopted in the CT26-^KrasG12C^ model (Figure 3A), including body weight (Supplementary Figure S4A) as well as subcutaneous and intracranial tumor growth (Figure 3B and C). Despite a reduction in tumor volumes in mice receiving anti-PD-1, in this model, it did not reach statistical significance compared to the vehicle group. Consistent with our results with CT26 cells, we again observed a complete remission of subcutaneous tumors in animals treated with both adagrasib alone or in combination with ICI (Figure 3B and Supplementary Figure S4B-E). Intracranially, a trend for a reduction in tumor growth was detected after 2 weeks of drug administration for both adagrasib and the combination groups compared to the control, which was statistically significant at 5 weeks (Figure 3C and Supplementary Figure S4F). In addition, anti-PD-1 treatment significantly controlled intracranial tumor growth compared to the vehicle (Figure 3C and Supplementary Figure S4F), but not to the extent observed for adagrasib and the combination groups.

**Figure 4.**
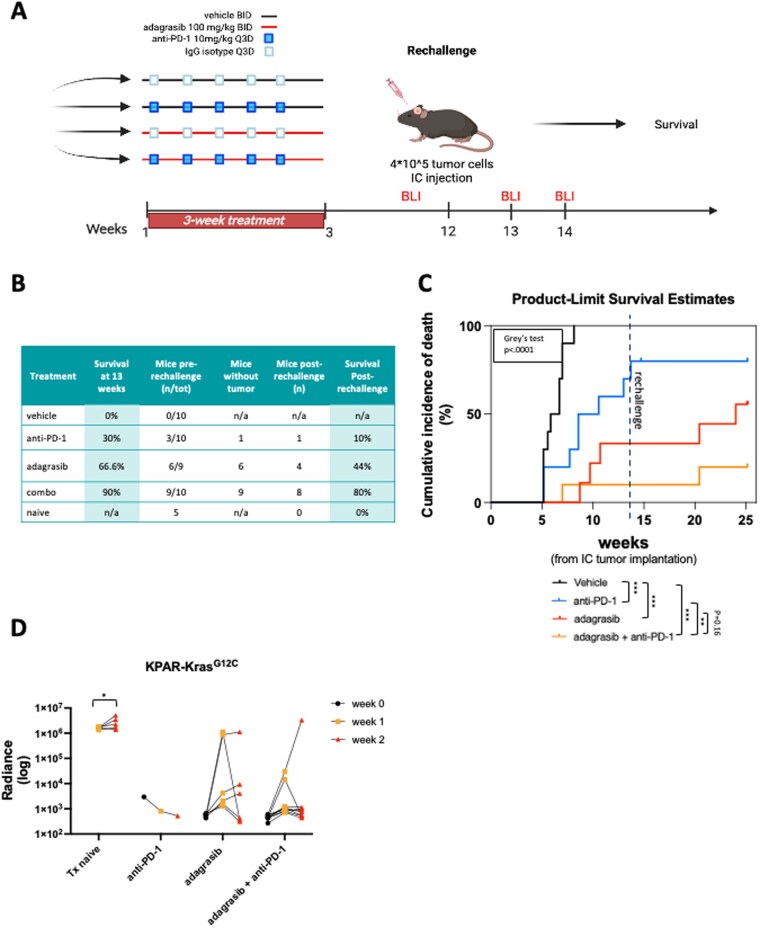
In vivo KPAR tumor rechallenge model. (A) Schematic of in vivo KPAR tumor rechallenge. Created in BioRender. Torrini, C. (2026) https://BioRender.com/9az0xjz. Survived animals from each group were rechallenged with a 5 times higher concentration of tumor cells and monitored for overall survival and tumor growth using bioluminescence imaging (BLI). (B) Table shows the census of surviving animals at 11 weeks (pre-rechallenge), mice enrolled in the rechallenge experiment as well as the survival post-rechallenge. (C) Cumulative incidence of death observed in KPAR mice upon KRASi therapy alone and in combination with anti-PD-1 (Grey’s test, *P* < .001). Pairwise comparisons were used with the Benjamini-Hochberg adjustment at FDR = 0.05 (*P* ≤ .001), *n* = 9/10 per group. Two of the three mice in the anti-PD-1 group developed tumor recurrence, leading to the exclusion of these animals from subsequent rechallenge experiments. Survival estimates allow for a competing risk of non-rechallenge due to intracranial tumor relapses. (D) Intracranial tumor volume was monitored at two time points post-rechallenge using BLI. Tx naïve: treatment naïve mice used as a positive control. Statistical analysis assessing the significance of the tumor volume at week 2 compared to week 1 after rechallenge for each group. Results of the two-way ANOVA analysis using Turkey’s multiple comparisons test are shown.

We conducted the long-term follow-up of the surviving animals (Figure 4A). At week 13, while none of the animals in the vehicle group survived, 3 out of 10 animals in the ICI group were still alive, while 6 out of 9 in the adagrasib group, and 9 out of 10 in the combination group were alive (Figure 4B). However, we observed tumor regrowth in 2 of the 3 mice in the anti-PD-1 group, showing that in this model, tumor recurrences occurred after immunotherapy discontinuation, resulting in the exclusion of these animals from subsequent rechallenge studies.

We rechallenged the remaining animals with a higher dose of KPAR lung cancer cells as previously done for the CT26 model and monitored intracranial tumor growth and overall survival for 11 weeks (Figure 4C and D and Supplementary Figure S5A). We observed a clear distinction in the overall survival between different treatments, as 10%, 44%, and 80% of animals in the anti-PD-1, adagrasib alone, and combination group, respectively, achieved long-term survival (Figure 4B and C). The cumulative incidence of death analysis, assessed using Gray’s test, showed that the combination treatment conferred a significant survival benefit compared with the vehicle and anti-PD-1 alone groups as well as a trend for a greater survival compared to single agent adagrasib (Figure 4C and Supplementary Figure S5B).

Taken together, these findings highlight the efficacy of this combination strategy in long-term preclinical models of BM harboring KRAS^G12C^ mutations.

To understand whether adagrasib and anti-PD-1 enhanced T cell killing activity, we used 48h co-cultures of activated T cells and CT26 or KPAR cells at two different target-to-effector ratios (1:1 and 1:100) in the presence of adagrasib alone or in combination with anti-PD-1 and evaluated tumor cell viability (Supplementary Figure S6A-C). Our data showed that in both CT26 and KPAR models, we observed a significant decrease in tumor cell viability in the co-cultures treated with adagrasib alone or in combination with anti-PD-1 (Supplementary Figure S6B and C). Of note, although we detected the highest T cell killing of CT26 cells in the combination treatment at the 1:100 tumor-T cell ratio (Supplementary Figure S6B), we did not observe this with KPAR cells (Supplementary Figure S6C). Since our in vivo data showed the benefit of the combination treatment in both models, these data suggest different mechanisms of action between models that may not be fully recapitulated and detected in tumor-T cell co-cultures in vitro.

## Discussion

BM are common in patients with advanced KRAS mutant lung cancer, and because the CNS is associated with unique immune and drug delivery challenges, their occurrence is associated with poor clinical outcomes.^22-24^ Over the past decade, KRAS-targeted therapies, such as the KRAS^G12C^ inhibitor, adagrasib, have been approved for treating solid tumors and are now being evaluated for BM. However, resistance and tumor recurrence commonly occur after treatment discontinuation, suggesting the need for combination strategies. Since the KRAS^G12C^ oncogenic mutation is known to promote tumor immune evasion with high levels of PD-L1,^13^ the combination of immunotherapy with KRAS-targeted therapy has been explored as a promising therapeutic approach in mouse models of extracranial disease,^4^ while its intracranial activity remains unexplored. In this study, we aimed to fill this gap, investigating the efficacy of this combination therapy in *in vivo* models of BM.

Although intracranial mouse models of BM have been instrumental in understanding solid tumor growth in the brain, these models may not recapitulate the BM immune microenvironment. The immune landscape of BM represents an ongoing challenge for in vivo studies. During metastatic cancer progression in humans, the host immune system has already been exposed to primary tumor antigens, thus making the traditional intracranial-only models not ideal for evaluating immunotherapies in the context of CNS cancer progression. Therefore, we created immunotypic BM models that better mimic the sequential exposure of the immune system, where we first implanted tumors subcutaneously and then intracranially, obtaining dual BM tumor models.^19^ In addition, it has been shown that because of the complexity of the BM microenvironment, intracranial ICI response may be primed by the presence of extracranial disease,^19^^,^^20^^,^^25-27^ underscoring the value of using dual-tumor BM models for evaluating combination therapies with anti-PD-1. Moreover, patients with BM are more likely to have a partial response to ICI compared to patients with primary brain tumors only. Using these dual-tumor models enables the study of ICI interventions in a system that may more accurately reflect the immunological context and clinical response of BM than intracranial implantation alone.

Herein, we demonstrate, for the first time, the intracranial efficacy of adagrasib and ICI combination using two KRAS^G12C^ dual tumor syngeneic mouse models, showing that combining adagrasib with anti-PD-1 immunotherapy can prevent BM recurrences originating from KRAS^G12C^ solid tumors. While in both BM models, we observed strong antitumor activity of adagrasib monotherapy, which effectively arrested subcutaneous tumor growth, we also reported model-specific responses to treatments. Specifically, in the colorectal BM model, both adagrasib and anti-PD-1 reduced intracranial tumor growth, and adagrasib significantly prolonged survival compared to vehicle. Importantly, in the lung cancer BM model established with KPAR cells, the combination treatment achieved a superior therapeutic response compared to monotherapy. These data are consistent with previous literature in the extracranial setting,^5^ and validate CNS penetrance and intracranial benefit of adagrasib in two different models, supporting the clinical relevance of our findings.

Another important consideration for BM patients treated with targeted therapy is the high risk of recurrence. In this context, we used tumor re-challenge experiments to mimic tumor recurrences. We demonstrated the benefit of combining KRAS targeted therapy and anti-PD-1 in preventing intracranial tumor recurrences for the long-term (up to 5 months of treatment discontinuation) in two models of BM. Although high response rates to the combination treatment were consistently noted across the two models, response to the single agents (anti-PD-1 and adagrasib only) was greater in the CT26 model. This observation may be due to differences in tumor immunogenicity and the tumor immune microenvironments (TIME) in the two models.^14^^,^^18^^,^^28^ Although both CT26 and KPAR cells were modified to have Kras^G12C^, they differ in genetic background and immune profile.^29^ Specifically, CT26 cells form tumors with high levels of immune infiltration characterized by TILs, such as CD8+T cells, Foxp3− CD4+T cells, and dendritic and NK cells.^30^ Consistent with our in vivo data, this tumor microenvironment likely renders this model more responsive to ICI.^30^^,^^31^ In contrast, KPAR cells, though highly immunogenic,^32^ engage in a broader immune response, characterized by an immune suppressive tumor microenvironment. This includes the presence of regulatory T cells and PD-L1+ myeloid cells, which promote immune evasion.^32^ The intrinsic ICI-resistant nature of KPAR tumors^18^ mirrors the immune-resistant profile often observed in BM and may explain its partial resistance to anti-PD-1 as single agent treatment, while showing encouraging response to the combination treatment. Of note, our in vitro data showed that adagrasib increased Cd274 (PD-L1) expression in KPAR cells, which may contribute to enhancing suppressive TIME and mediating an improved response to anti-PD-1. The efficacy of the combination treatment is likely due to a dual effect of adagrasib on immune and tumor cells. Adagrasib can block tumor cells from producing cytokines, arresting the recruitment of immune suppressive cell population, while promoting the recruitment of NK cells and activating cytotoxic T cells able to respond to anti-PD-1 effectively. Because the KRAS mutation induces a high expression of PD-L1^9^ and the overload of PD-1-PD-L1 interactions can overwhelm ICI alone, adagrasib-mediated reduction of PD-L1 levels on tumor cells may make ICI effective. Nevertheless, our findings suggest that pharmacological modulation of BM immune system may be needed for achieving a complete tumor remission and preventing recurrences. However, our investigation is limited by its focus on testing combination therapy for BM recurrence. Future work should explore the detailed mechanisms by which pharmacological targeting of the BM immune system influences tumor remission and relapse.

Treatment-induced reshaping of the TIME was characterized recently in preclinical extracranial models of lung cancer using spatial multiplex analysis.^33^ Our work represents a strong rationale for pursuing similar investigations intracranially. Such studies would help define the specific immune populations that mediate treatment-driven tumor clearance and clarify how single-agent or combination therapies affect these populations, using approaches such as flow cytometry and/or single-cell analysis.

Collectively, our data describe the benefit of using adagrasib in combination with anti-PD-1 immunotherapy as a therapeutic strategy to control growth and prevent recurrences of BM in two preclinical models of BM. Given the high intracranial recurrence rates in patients with BM and the limited effective treatment options, our work holds potential clinical relevance for this patient population. Our data support ongoing clinical investigations testing adagrasib in combination with pembrolizumab in KRAS-mutant lung cancer BM patients (KRYSTAL-7),^34^^,^^35^ thus offering new hope for the use of CNS-penetrant targeted therapies combined with ICI in a patient population that has been traditionally excluded from clinical trials.

## Supplementary Material

vdag107_Supplementary_Data

## Data Availability

The overall data generated in these studies will be publicly available to the scientific community upon reasonable request.
