# Supplementary material for: Combining the KRASG12C inhibitor adagrasib with anti-PD-1 immunotherapy improves overall survival and prevents recurrence in preclinical models of brain metastasis
Source: Neurooncol Adv. 2026 Jun 2;8(1):vdag107. doi: 10.1093/noajnl/vdag107 (PMC13242296; doi:10.1093/noajnl/vdag107)
Supplement: vdag107_Supplementary_Data [file vdag107_supplementary_data.zip › Torrini et al Neuro-Onc Advances Supplement_Revised_Final.docx]

**Supplementary Figure 1: Gene expression changes induced by adagrasib in KRAS-mutant cancer cells.** CT26 colorectal cancer cells (**A**) and KPAR lung cancer cells (**B**) were treated with 500 nM and 1 μM adagrasib for 24h. Expression of genes related to KRAS-dependent MAPK targets, MHC class I antigen-presenting machinery, cytokines, and immune suppressive tumor microenvironment, was evaluated using RT-qPCR. Data were normalized over Rpl13a gene expression and shown in fold changes (FC) over control (DMSO). A two-way ANOVA test was used for statistical analysis.

**Supplementary Figure 2: Drug tolerability and intracranial treatment response in the CT26 model.** **A:** Animal body weight was monitored during three-week treatments to assess tolerability of the KRASi, adagrasib and/or anti-PD-1 immune therapy or vehicle (control). n=10 per experimental group. **B-E:** Subcutaneous tumor volume measurements over time are shown for each group. Time is shown as days after intracranial tumor injection**.** **F:** Bioluminescence images are shown at 3 time points: pre-treatment, two mid-treatment (week 1 and week 2 after treatment initiation), and (**G**) post-treatment at 3 weeks after treatment discontinuation.

**Supplementary Figure 3: CT26 tumor rechallenge monitoring. A:** Bioluminescence images are shown at 3 time points: pre-rechallenge (week 0), and at two post-rechallenge time points (week 1 and week 2). **B:** Survival probability for the CT26 rechallenged mice is shown, including the number of subjects at risk over time. Naïve= tumor naïve mice were used as a positive control for tumor growth.

**Supplementary Figure 4: Drug tolerability and intracranial treatment response in the KPAR model.** **A:** Animal body weight was monitored during three-week treatments to assess the tolerability of the KRASi, adagrasib and/or anti-PD1 immune therapy or vehicle (control). n=9-10 per experimental group. **B-E:** Subcutaneous tumor volume is shown over time for individual experimental groups. **F:** Bioluminescence images are shown at 3 time points: pre-treatment, two mid-treatment (week 1 and week 2 after treatment initiation).

**Supplementary Figure 5: KPAR tumor rechallenge monitoring. A:** Bioluminescence images are shown at 3 time points: pre-rechallenge (week 0), and at two post-rechallenge time points (week 1 and week 2). **B:** Overall survival for the KPAR rechallenged mice is shown, including the number of subjects at risk over time. Naïve= tumor naïve mice were used as a positive control for tumor growth.

**Supplementary Figure 6: T cell killing Assay. A:** Schematic of the experimental design is shown. Images were created using BioRender template. Briefly, T cells were isolated from Balb/c or C57bl/6 mouse spleens, activated by CD3 and CD28 coating overnight on 6-well plates, and followed by 7 days of expansion. Then, respective T cells were added to CT26 or KPAR FmC-tagged tumor cells, with anti-PD-1 (10 µg/mL), adagrasib (500 nM), or their combination. IgG and DMSO were used for the control group. Tumor cell viability was read at 48h by luminescence. **B and C:** Cell viability of CT26 colorectal cancer (**B**) or KPAR lung cancer cells (**C**) after co-culture with two different concentrations of T cells is shown. Two-way ANOVA, multi-comparison analysis was used for statistical analysis (n=4 replicates/ group).

**Supplementary Material**

**Cell Culture** KPAR cells were cultured in high glucose DMEM (Gibco, Cat# 10-013-CV), in the presence of 10% the fetal bovine serum (Gibco, Cat# A3160402) and antibiotic/antimycotic, (Cat# 15240062, Gibco) and maintained in an incubator at 37°C with 5% CO_2_. While CT26 cells required RPMI-16-40 (ATCC, 30-2001). All cells were pathogen tested using the VRL CytoClear Premium panel and periodically assessed for mycoplasma-free condition.

**Gene Expression** RNA isolation was performed using the Norgen RNA/DNA Purification Kit (SKU:48700). cDNA was obtained using the High-Capacity RNA-to-cDNA™ Kit (Thermo Fisher Scientific, cat# 4387406). The following mouse TaqMan probes were used: cd274 (Mm03048248_m1), B2m (Mm00437762_m1), Ifnb1 (Mm00439552_s1), Axl (Mm00437221_m1), Tap1(Mm00443188_m1), Cxcl10 (Mm00445235_m1), Dusp4 (Mm0072376_m1), Evt4 (Mm00476696_m1) and Rpl13a (Mm05910660_g1) was used as housekeeping. Gene expression is shown as fold changes over the DMSO control.

**Dose-response assay** Cell viability was measured using the CellTiter Glo 2.0 kit (Promega Cat#G9241).

**Drug Administration** Adagrasib powder was resuspended in 10% Captisol and 50 mmol/L citrate buffer pH 5.0 (vehicle) and stored at 4°C, according to Mirati Inc guidelines. Anti-PD-1 antibodies used was purchased from BioXcell (Cat# BE0146), as well as IgG control (Cat# BE0089)
