# Supplementary figures and images for: Combining the KRASG12C inhibitor adagrasib with anti-PD-1 immunotherapy improves overall survival and prevents recurrence in preclinical models of brain metastasis
Source: Neurooncol Adv. 2026 Jun 2;8(1):vdag107. doi: 10.1093/noajnl/vdag107 (PMC13242296; doi:10.1093/noajnl/vdag107)

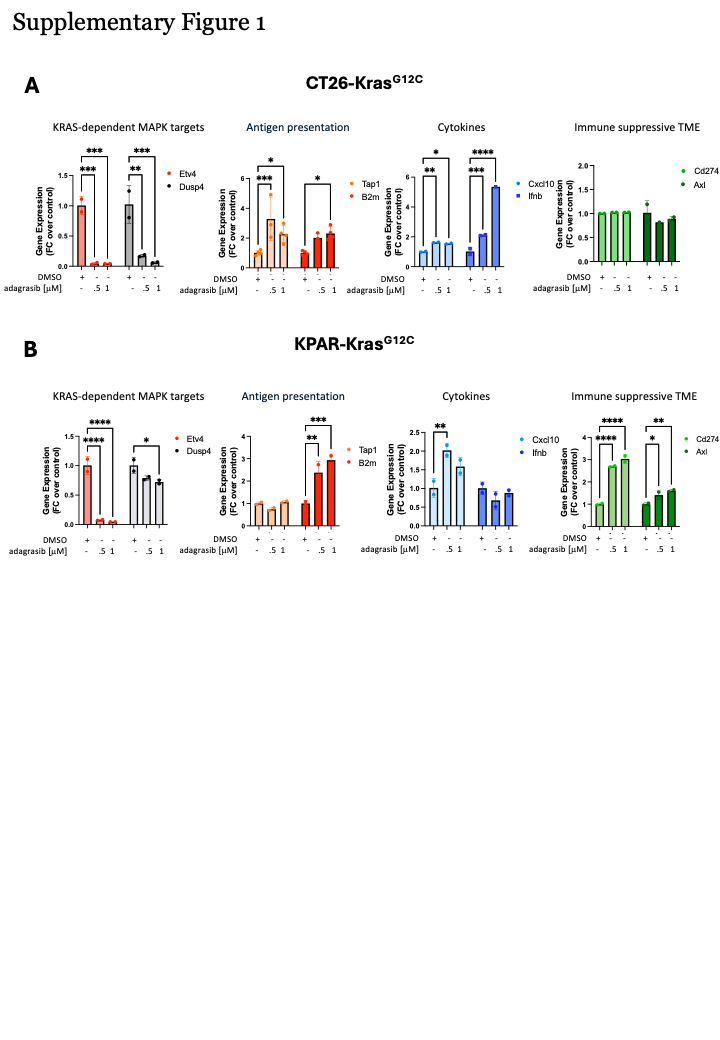

Supplement: vdag107_Supplementary_Data [file vdag107_supplementary_data.zip › Supplementary Figure 1.tiff]

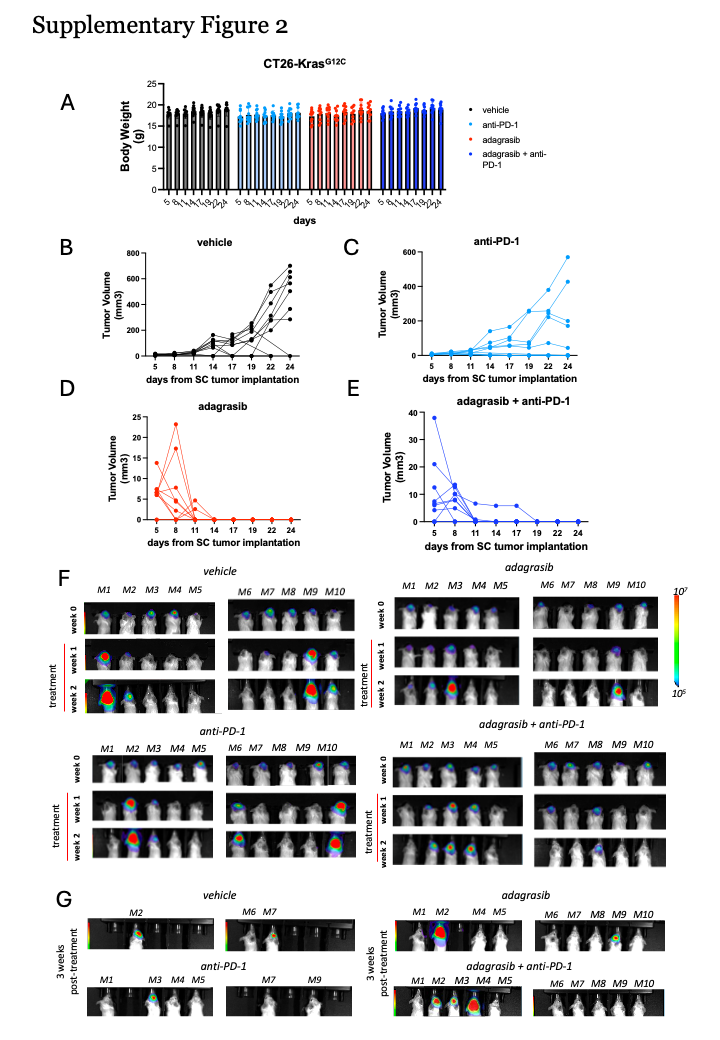

Supplement: vdag107_Supplementary_Data [file vdag107_supplementary_data.zip › Supplementary Figure 2.tiff]

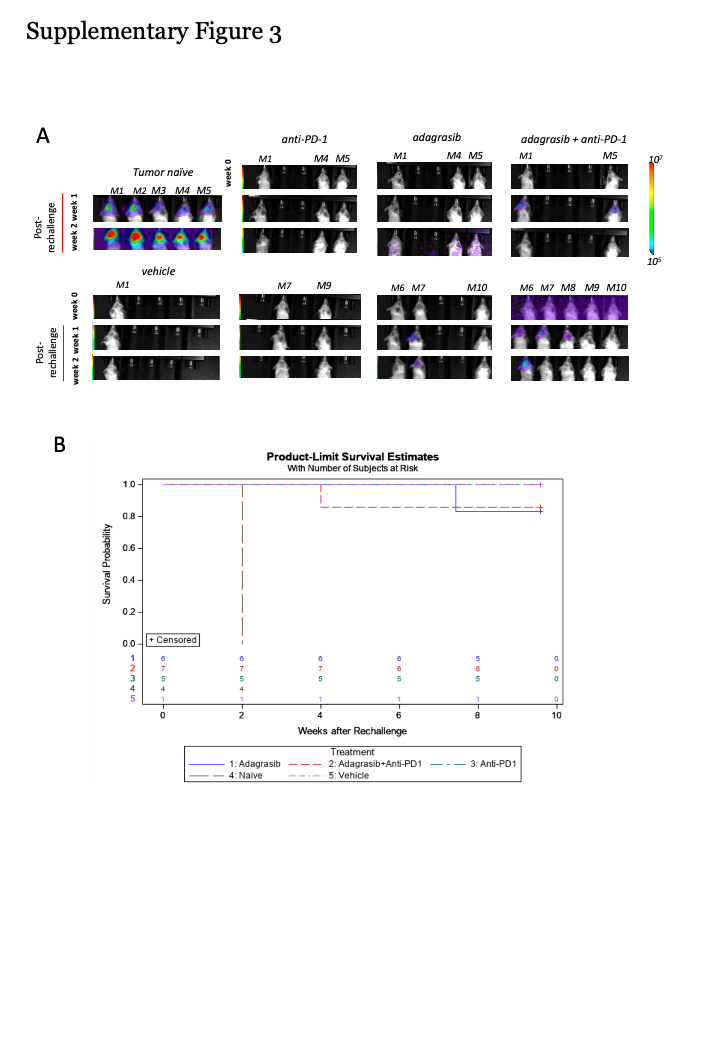

Supplement: vdag107_Supplementary_Data [file vdag107_supplementary_data.zip › Supplementary Figure 3.tiff]

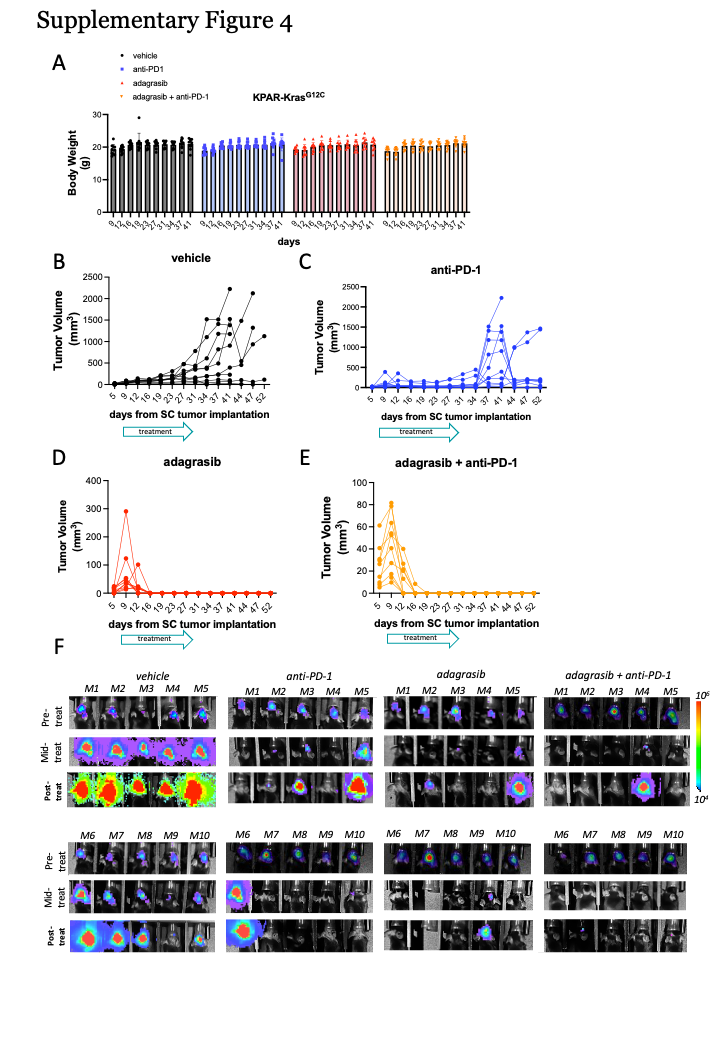

Supplement: vdag107_Supplementary_Data [file vdag107_supplementary_data.zip › Supplementary Figure 4.tiff]

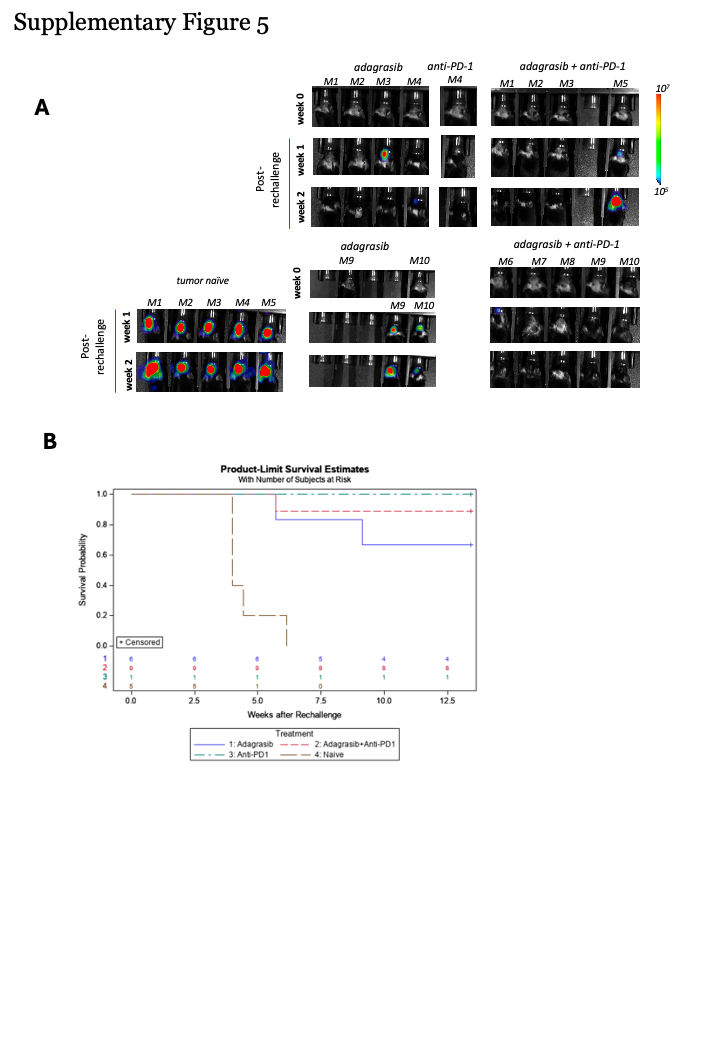

Supplement: vdag107_Supplementary_Data [file vdag107_supplementary_data.zip › Supplementary Figure 5.tiff]

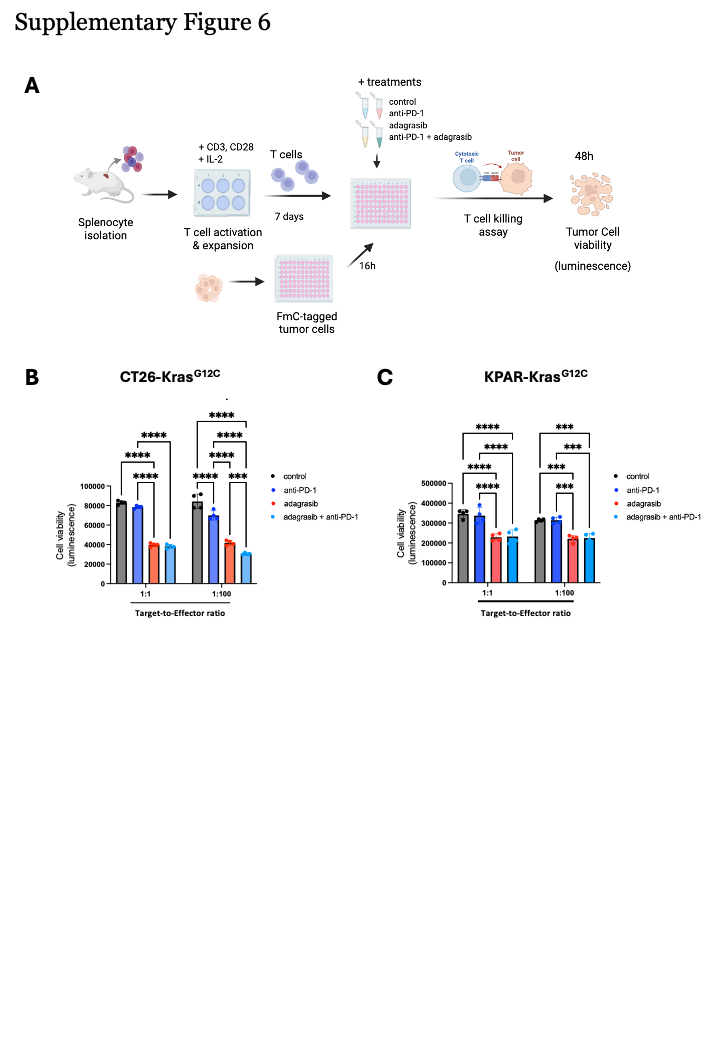

Supplement: vdag107_Supplementary_Data [file vdag107_supplementary_data.zip › Supplementary Figure 6.tiff]
